# Cancer Epidemiology and Decentralization Service Program Initiation: A Cross-Sectional Analysis of Hospital-Based Oncology Admissions and Cancer Service Disparities

**DOI:** 10.3390/curroncol33090552

**Published:** 2026-09-11

**Authors:** Zukiswa Jafta, Muambangu Jean Paul Milambo, Wilson Wezile Chitha

**Affiliations:** 1 Cancer Research & Innovation Platform of the Eastern Cape (CRIPEC), Division of Oncology, Walter Sisulu University, Mthatha 5117, Eastern Cape, South Africa; 2 WSU Institute for Clinical Governance and Healthcare Administration, School of Public Health, Walter Sisulu University, Mthatha 5117, Eastern Cape, South Africa

**Keywords:** cancer, health system decentralization, service utilisation, disparities, South Africa

## Abstract

**Simple Summary:**

Cervical cancer was the dominant gynaecological malignancy (82.5%), with most patients presenting at advanced stage (62.7% Stage III), reflecting delayed diagnosis in the Eastern Cape. HIV co-infection was common (57.3%) and significantly associated with advanced stage (*p* < 0.001), highlighting a strong infectious oncology endemic. Treatment was primarily radiotherapy (83.2%), with very limited surgery (<2%) due to late presentation. Despite system constraints, treatment completion was high (86.6%) across all stages (*p* = 0.824), indicating service delivery resilience. However, patients experienced substantial diagnostic and treatment delays, contributing to advanced disease at presentation. Quality of life declined markedly with progression, from 85 ± 5 (Stage I) to 25 ± 7 (Stage IV). The findings underscore the need for improved early detection, faster diagnostic pathways, and integrated HIV–cancer care.

**Abstract:**

Background: Cancer remains a growing public health challenge globally, with increasing demand for oncology services, particularly in resource-limited settings. In South Africa’s Eastern Cape Province, tertiary hospitals face rising patient loads amid constrained infrastructure and late-stage presentation. This study assessed temporal trends, gender differences, and cancer-type distribution in oncology service utilisation at Nelson Mandela Academic Hospital (NMAH) from April 2023 to February 2025. Methods: A retrospective quantitative observational time-series design was used. Monthly aggregated oncology service data were extracted from hospital records, including total cancer attendances, gender distribution, cancer types, and service utilisation indicators (new cases, follow-ups, chemotherapy, hormone therapy, palliative care, admissions, referrals, and mortality). Descriptive statistics, Pearson correlation, and simple linear regression were applied to assess temporal trends. Time (months) was the independent variable. Results: A total of 16,368 cancer-related encounters were recorded, with a mean of 706.4 cases per month. Females accounted for 11,816 cases, significantly exceeding males (4552). Breast cancer was the most prevalent malignancy (5763 cases), followed by other cancers (4316) and cervical cancer (3616). Cervical cancer demonstrated a strong and statistically significant increasing trend (r = 0.619, *p* = 0.002), while lung cancer also showed a significant upward trend (r = 0.549, *p* = 0.007). Breast cancer showed a positive but non-significant trend (*p* = 0.163), whereas prostate and oesophageal cancers showed no significant temporal changes. Service utilisation was dominated by follow-up visits (14,761) and palliative care (16,911), indicating a high burden of advanced disease. Overall attendance showed an increasing trend, peaking at 1039 cases in April 2024, with seasonal declines in December–January. Conclusions: Oncology service utilisation at NMAH is increasing, with marked gender disparities and rising trends in cervical and lung cancers. The high reliance on palliative and follow-up care highlights late presentation and advanced disease burden. Strengthening early detection, screening programmes, and decentralised oncology service capacity is essential to address the growing cancer burden in this setting.

## 1. Background

Cancer remains a major global public health challenge and is among the leading causes of morbidity and mortality worldwide, with a continuously rising burden driven by demographic transitions, environmental exposures, and lifestyle factors [1,2]. Health systems globally are under increasing pressure to expand oncology services, including prevention, diagnosis, treatment, and palliative care [2]. In the United States, cancer surveillance is strengthened through systems such as the SEER programme, which provides detailed population-level data on incidence, survival, and mortality trends. Despite these advanced systems, significant disparities persist in access to early detection and treatment across different population groups [3]. In Australia, structured national screening programmes have contributed to improved survival outcomes for major cancers. However, inequalities continue to affect rural populations and Indigenous communities, who experience delayed diagnosis and reduced access to specialist oncology services [4].

Canada benefits from universal healthcare coverage and robust cancer surveillance systems, yet geographic disparities remain a challenge, particularly in remote and northern regions where access to oncology services is limited [5]. Asia carries a substantial proportion of the global cancer burden, with lung, liver, gastric, and colorectal cancers being the most common. Rapid urbanisation, tobacco use, dietary changes, and environmental pollution are major contributing factors to this increasing burden [1,6].

In Africa, cancer incidence is rising rapidly, with late presentation and limited access to treatment contributing to poor outcomes. Cervical, breast, and prostate cancers remain the most common malignancies across many settings [7,8]. South Africa faces a dual burden of communicable and non-communicable diseases, with increasing cancer incidence placing additional strain on the health system. Inequalities in access to oncology services persist between the public and private healthcare sectors [7]. The Eastern Cape Province experiences significant oncology service constraints due to limited infrastructure and concentration of specialist services in tertiary hospitals. Late presentation of cancer cases and weak referral systems further contribute to poor outcomes [7,8].

Nelson Mandela Academic Hospital serves as a key tertiary oncology referral centre in the Eastern Cape, managing increasing patient volumes under resource-constrained conditions. This creates a need for continuous monitoring of service utilization trends to support planning and resource allocation [7].

Across all regions, a consistent pattern emerges of rising cancer burden, unequal access to care, and increasing pressure on health systems, highlighting the need for strengthened data-driven oncology service planning [1,6]. Cancer service demand in South African public hospitals, particularly in the Eastern Cape, continues to increase, yet there is limited longitudinal evidence describing oncology service utilization patterns at the facility level. Understanding temporal trends, gender differences, service correlations, and cancer-type distribution is essential for strengthening planning, improving efficiency, and supporting equitable resource allocation at Nelson Mandela Academic Hospital. This study aimed to analyse temporal trends, gender differences, oncology service utilization patterns, and cancer-type distribution among patients attending oncology services at Nelson Mandela Academic Hospital between April 2023 and February 2025.

## 2. Methods

### 2.1. Study Design and Setting

This study adopted a retrospective quantitative observational time-series design to examine cancer care utilization trends at Nelson Mandela Academic Hospital (NMAH), Eastern Cape, South Africa. The hospital is a tertiary referral and teaching institution providing oncology services to a largely rural and socioeconomically disadvantaged population. In such low- and middle-income health systems, tertiary facilities often experience increasing oncology demand due to late presentation, limited screening coverage, and constrained primary care oncology capacity [9,10].

### 2.2. Data Collection, Management, and Analysis

Monthly aggregated oncology service data from April 2023 to February 2025 were extracted from hospital records. Variables included total cancer attendance, gender distribution, cancer-type categories, and service indicators such as new cases, follow-ups, chemotherapy, hormone therapy, palliative care, inpatient admissions, and mortality. Data represented service utilization rather than confirmed incidence cases, consistent with hospital-based cancer monitoring approaches [1,10]. Data were cleaned, coded, and entered into a secure statistical database with the removal of any patient identifiers to ensure confidentiality.

Data analysis was performed using descriptive statistics, Pearson correlation analysis, and simple linear regression models. Time (months) was used as the independent variable to assess trends in cancer service utilization. Regression outputs included beta coefficients, *p*-values, confidence intervals, and R^2^ values, while graphical analysis was used to visualize temporal and seasonal patterns. This approach is widely applied in global cancer surveillance and health systems research to assess trends in service delivery and burden changes over time [2,9,11,12,13,14].

### 2.3. Ethical Considerations

This study was approved by the Walter Sisulu University Research Ethics Committee (WSU-REC) on 11 March 2026 under approval number REC-120209020 and WSUHRECO40/2026. Ethical approval was obtained for the use of de-identified patient data from hospital records in accordance with the ethical standards set by Walter Sisulu University and the Eastern Cape Department of Health. Informed consent was not required for this retrospective observational study, as it involved the use of aggregated data that did not identify individual patients. All procedures were carried out in line with ethical guidelines for medical research and data protection laws. Permission to access institutional data was granted by Nelson Mandela Academic Hospital management. The study used anonymized, aggregated secondary data, ensuring that no patient identifiers were included. All procedures complied with ethical standards for secondary health data research as recommended by the World Health Organization for health systems and cancer surveillance studies [15]. Confidentiality, data protection, and responsible use of institutional records were strictly maintained throughout the study.

## 3. Results

### Overview of Cancer Service Utilisation

The study analysed oncology service utilisation at Nelson Mandela Academic Hospital (NMAH) from April 2023 to February 2025. The findings demonstrate an overall increasing cancer burden, marked gender disparities, and heterogeneous trends across different cancer types. Results are presented using descriptive statistics, time-series analyses, and regression modelling to evaluate temporal changes in service demand. Table 1 summarises cancer-specific burden and temporal trends. Breast cancer accounted for the highest total caseload (5763 cases; mean 250.6/month), followed by other cancers (4316 cases; mean 187.7/month) and cervical cancer (3616 cases; mean 157.2/month). Cervical cancer showed a strong and statistically significant increasing trend (r = 0.619, *p* = 0.002), while lung cancer also demonstrated a significant upward trend (r = 0.549, *p* = 0.007). Breast cancer showed a positive but non-significant trend (*p* = 0.163). Prostate and oesophageal cancers showed no statistically significant temporal changes, indicating relatively stable incidence patterns over time.

**Figure 1:**
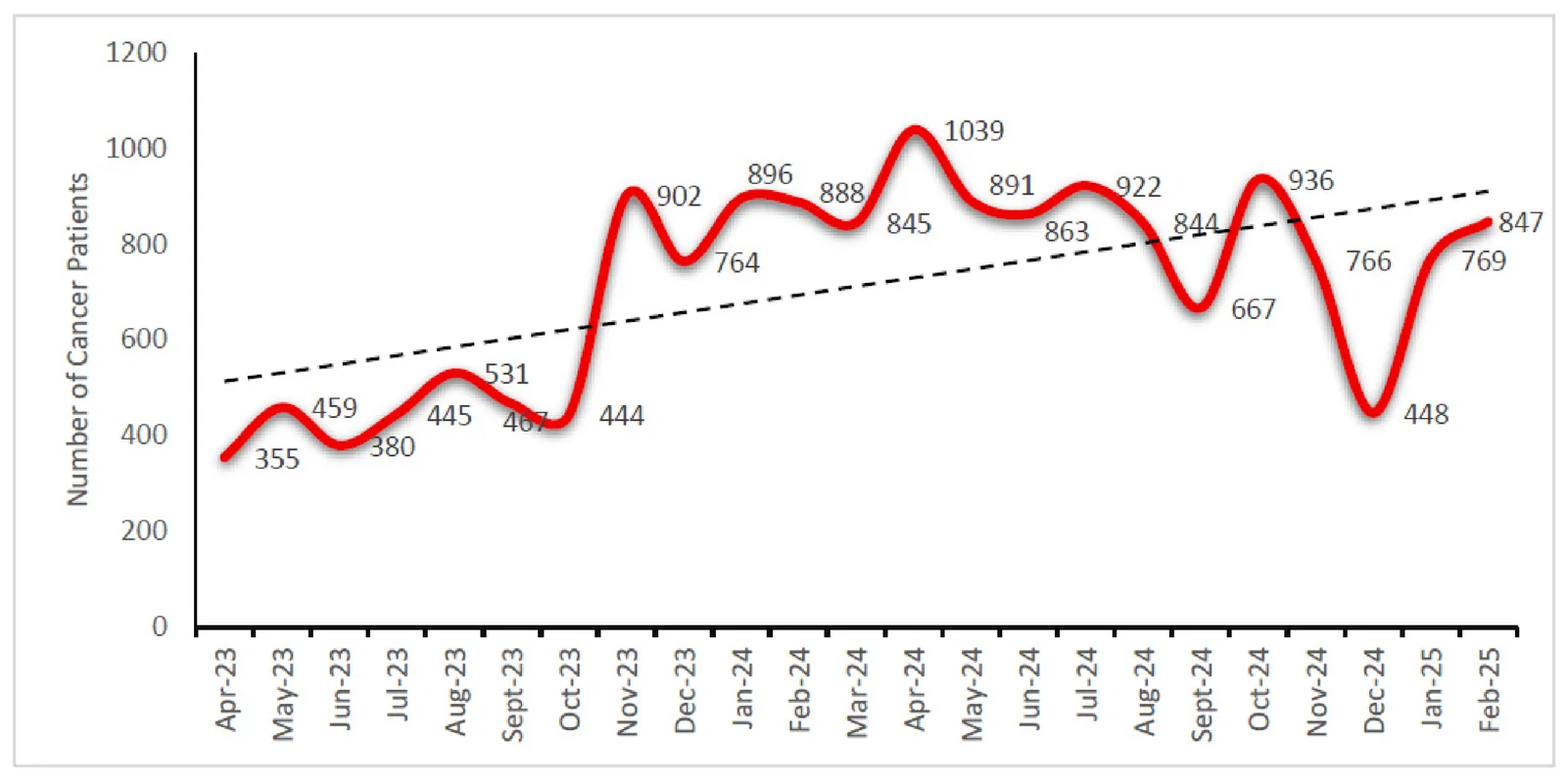
All graphs were generated using Microsoft Excel based on the same dataset presented in the corresponding tables. The figures visually represent the tabulated results for ease of interpretation and comparison. The left Y-axis represents the primary scale for the number of cancer cases and service utilization counts, while the right Y-axis represents secondary scaled variables where applicable (e.g., comparative trends or rate-based indicators). The X-axis represents the study period (April 2023 to February 2025), showing monthly distribution and trends in oncology service utilization.

Figure 1 illustrates overall oncology attendance trends, showing a progressive increase in patient volumes from April 2023, reaching a peak of 1039 cases in April 2024. Intermittent fluctuations were observed, with secondary peaks in November 2023 and October 2024. A decline was recorded in December 2024 and January 2025, likely reflecting seasonal service interruptions and reduced healthcare utilisation during holiday periods. Overall, the trend indicates a sustained increase in oncology service demand over time. Figure 1 illustrates the monthly trend in the number of cancer patients attending the oncology service at Nelson Mandela Academic Hospital from April 2023 to February 2025, and statistical outputs.

During the period April 2023 to February 2025, NMAH managed a substantial and fluctuating oncology workload, with a total of 16,368 cancer cases (mean: 706/month; range: 355–1039). Female patients constituted most cases (11,816) compared to males (4552). Service utilization was dominated by palliative care services (16,911 cases; mean: 735/month) and follow-up visits (14,761 cases; mean: 643/month), indicating a large proportion of patients requiring long-term and supportive care. New cancer cases averaged 93 per month, while chemotherapy (3864 total; 168/month) and hormone therapy (893 total; 39/month) reflected comparatively lower levels of curative or disease-modifying treatment. Health service utilization remained high, with 11,019 inpatient admissions (mean: 479/month), while referrals (2763 total; 120/month) showed notable variability across the study period. A total of 62 deaths were recorded (mean: 2.7/month; range: 0–7), indicating relatively low absolute mortality despite a high patient burden and constrained resources. Table 2 provides the statistics of cancer patients and oncology service utilization at NMAH (April 2023–February 2025).

**Figure 2:**
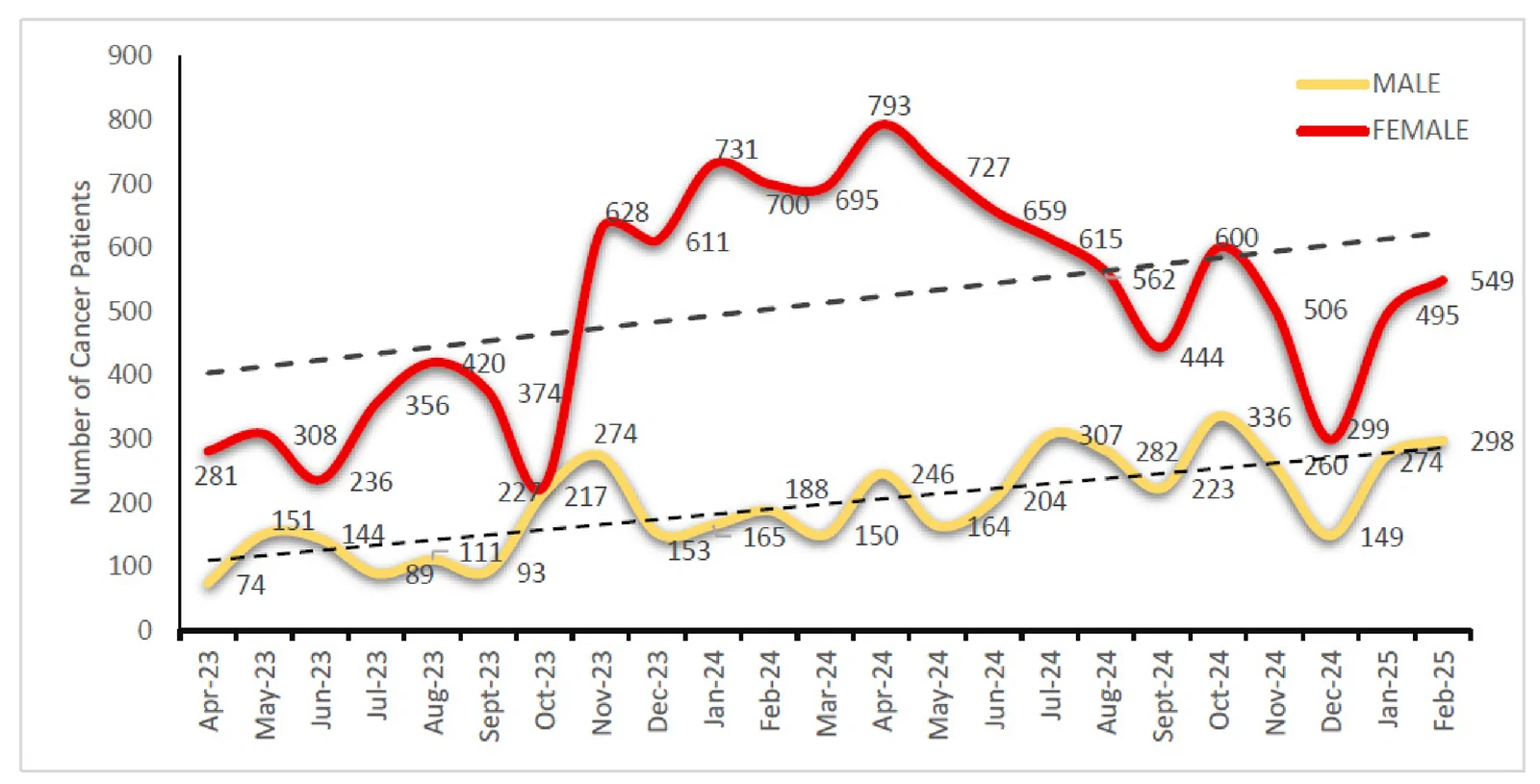
Monthly trend in cancer patient attendance at Nelson Mandela Academic Hospital by gender (Apr 2023–Feb 2025). All graphs were generated using Microsoft Excel based on the same dataset presented in the corresponding tables. The figures visually represent the tabulated results for ease of interpretation and comparison. The left Y-axis represents the primary scale for the number of cancer cases and service utilization counts, while the right Y-axis represents secondary scaled variables where applicable (e.g., comparative trends or rate-based indicators). The X-axis represents the study period (April 2023 to February 2025), showing monthly distribution and trends in oncology service utilization.

Figure 2 demonstrates persistent female predominance in cancer attendance throughout the study period. Female cases peaked at 793 in April 2024, while male cases reached a maximum of 336 in October 2024. Although both genders showed increasing trends over time, the rise among females was more pronounced. Noticeable fluctuations in both groups were observed during the December–January period, suggesting seasonal variation in healthcare utilisation and service access patterns.

**Figure 3:**
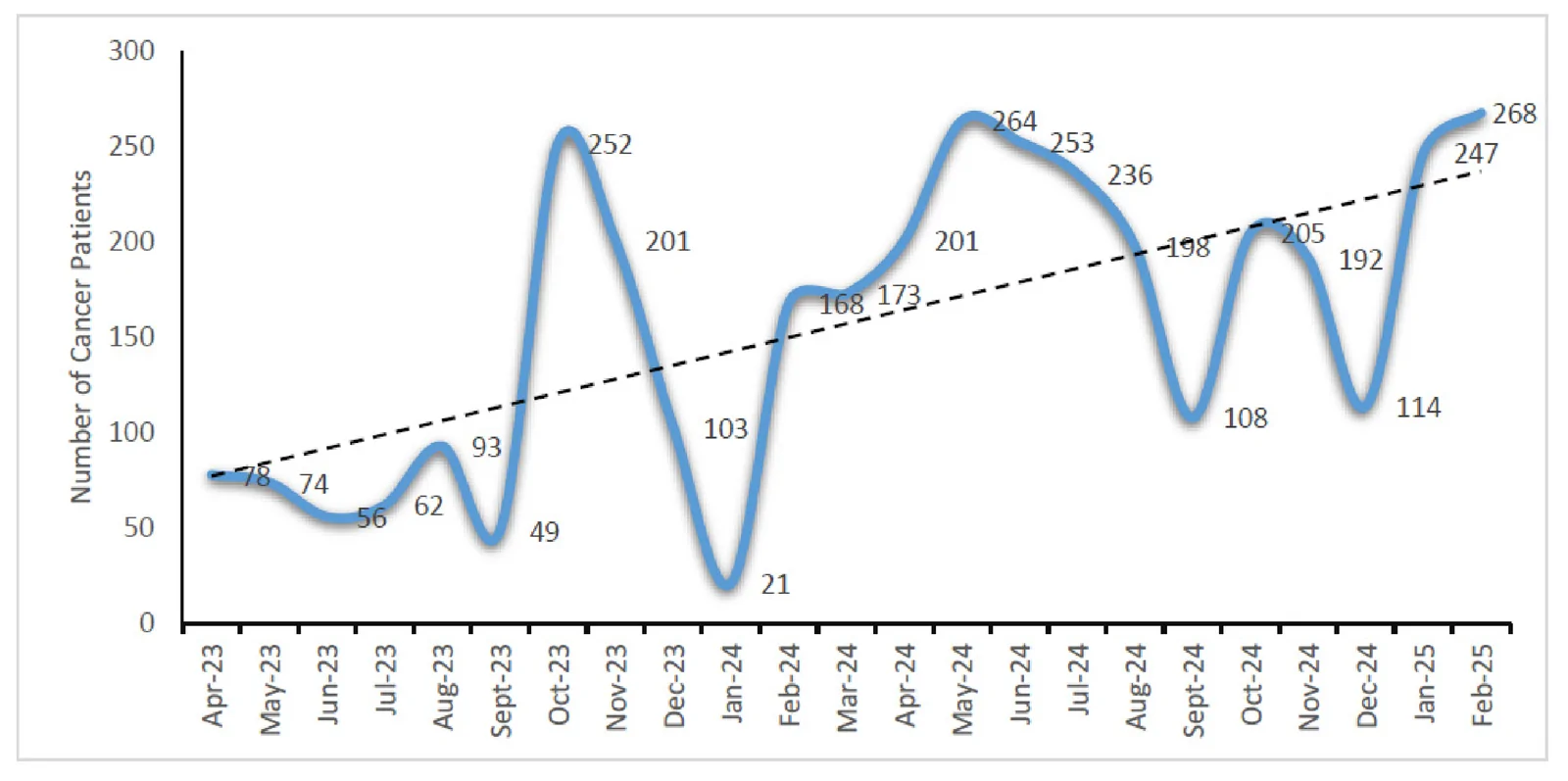
Monthly cervical cancer case trend at NMAH (April 2023–February 2025). All graphs were generated using Microsoft Excel based on the same dataset presented in the corresponding tables. The figures visually represent the tabulated results for ease of interpretation and comparison. The left Y-axis represents the primary scale for the number of cancer cases and service utilization counts, while the right Y-axis represents secondary scaled variables where applicable (e.g., comparative trends or rate-based indicators). The X-axis represents the study period (April 2023 to February 2025), showing monthly distribution and trends in oncology service utilization.

Figure 3 shows a consistent upward trend in cervical cancer cases, increasing from 78 cases in April 2023 to 268 cases in February 2025. Despite short-term fluctuations, the overall trajectory indicates a sustained increase in cervical cancer burden. This pattern highlights the growing need for enhanced screening, early detection, and strengthened HPV vaccination programmes.

The line graph displays the monthly trend in cervical cancer cases from April 2023 to February 2025. Although the number of cases fluctuates considerably across the months, a clear upward trend is evident, as shown by the linear trend line. The data indicate that cervical cancer cases increased over the period, rising from 78 cases in April 2023 to a peak of 268 cases in February 2025. The relatively low baseline of lung cancer cases in the early study period was followed by a gradual increase and a sharp peak of 79 cases in October 2024. This surge may reflect delayed diagnosis, cumulative case detection, environmental exposure risks, or improved diagnostic capacity within the facility. The variability suggests an unstable but rising burden over time. Lung cancer incidence at NMAH remained relatively low in the first half of the review period but rose gradually, culminating in a dramatic peak of 79 cases in October 2024. This rise may be linked to increasing tobacco use, environmental exposures, or late presentation due to non-specific early symptoms. The sharp spike may also reflect delayed diagnoses accumulating or improved diagnostic capacity (Table 1).

## 4. Discussion

The present study demonstrates a sustained increase in cancer care utilization at Nelson Mandela Academic Hospital (NMAH) between April 2023 and February 2025, with an average monthly upward trend of approximately 18 additional cases. Female patients consistently outnumbered males, while cervical and lung cancers showed statistically significant increasing trends. Strong correlations between new cases, follow-up visits, palliative care, and admissions indicate an integrated oncology service pathway responding to rising clinical demand. These findings reflect a growing oncology burden in the Eastern Cape, consistent with global projections of increasing cancer incidence and health system pressure [1,2].

When compared with other South African provinces, the Eastern Cape continues to experience disproportionate oncology service constraints, largely due to limited screening coverage, delayed referrals, and centralised specialist services. National cancer control strategies emphasize early detection and equitable access; however, implementation gaps persist, particularly in rural provinces. The increasing burden of cervical cancer aligns with known patterns in sub-Saharan Africa, where screening coverage remains suboptimal despite the availability of preventive strategies such as HPV vaccination [7,12]. The observed rise in breast cancer service utilization also mirrors national trends, where breast cancer remains the leading malignancy among women [13,14,15].

At a continental level, similar patterns of late presentation and rising cancer incidence have been reported across African countries, including Kenya, Nigeria, and Ghana, where health systems face comparable challenges in diagnostic capacity and oncology workforce shortages. In contrast, high-income countries such as the United States, Australia, and Canada demonstrate improved survival outcomes due to structured screening programmes, robust cancer registries, and timely access to treatment [3,4,5]. Organisations such as the American Cancer Society (ACS) and the American Society of Clinical Oncology (ASCO) emphasize early detection, multidisciplinary care, and precision oncology as key pillars of cancer control, approaches that remain under-resourced in many African settings [6].

Globally, cancer care is shifting toward precision medicine, genomic profiling, and personalised treatment strategies; however, such advancements remain limited in resource-constrained settings. The current findings highlight the need for health system strengthening before advanced technologies can be fully integrated. The strong linkage between service indicators (follow-ups, admissions, and palliative care) suggests increasing pressure on downstream oncology services, reinforcing the importance of upstream prevention and early detection strategies [1,10].

## 5. Strengths and Limitations

A key strength of this study is the use of longitudinal, real-world hospital data spanning 23 months, which enabled a detailed assessment of temporal trends in oncology service utilisation, gender differences, and service delivery patterns. The application of regression modelling and correlation analysis further strengthened the study by providing robust quantitative insights into oncology service dynamics over time.

However, several limitations should be acknowledged. The study relied on aggregated administrative data, which limited the ability to distinguish between new cancer diagnoses and repeat visits. In addition, the absence of clinical variables such as cancer staging, treatment response, and survival outcomes constrained deeper clinical interpretation. Furthermore, as the study was conducted in a single tertiary hospital, the findings may not be fully generalisable to broader provincial or national cancer burden patterns.

## 6. Clinical and Public Health Implications

The findings highlight an urgent need to strengthen early cancer detection and screening programmes, particularly for high-burden cancers such as cervical and breast cancer. The predominance of advanced disease presentations, reflected by high palliative care utilisation, underscores gaps in early diagnosis and timely referral.

From a preventive perspective, integrating human papillomavirus (HPV) vaccination, tobacco control strategies, and community-based cancer awareness programmes could substantially reduce the future cancer burden. Evidence from high-income settings demonstrates the effectiveness of HPV vaccination in reducing cervical cancer mortality trends over time [15]. Strengthening clinical pathways for early identification and referral is also critical to improving patient outcomes.

## 7. Health System and Policy Implications

At the health system level, the results support decentralising oncology services to district and regional hospitals to improve access, reduce care delays, and mitigate late-stage presentation. Enhancing primary healthcare capacity for cancer screening and early detection should be prioritised as part of universal health coverage strategies. Policymakers should also focus on improving referral systems and integrating digital health solutions to strengthen continuity of care, patient tracking, and information sharing across levels of care. Collectively, these interventions are essential for building a more responsive and equitable oncology care system capable of addressing the rising cancer burden.

## 8. Conclusions

The findings of this study indicate a steady increase in oncology service utilization at Nelson Mandela Academic Hospital, reflecting a growing cancer burden with important implications for service delivery, health system planning, and public health policy. The observed gender disparity, with a higher burden of cancer among women, highlights the need for targeted, gender-sensitive cancer prevention and control strategies. The increasing trends in cervical and lung cancers, together with the predominance of palliative care services, suggest that many patients continue to present at advanced stages of disease, underscoring critical gaps in early detection and timely intervention. These findings emphasize the urgent need to strengthen and expand cancer screening programmes, particularly for cervical and breast cancers, while also improving community-level awareness and health-seeking behaviour. In addition, integration of HPV vaccination, tobacco control measures, and community-based health education programmes is essential to reduce future cancer incidence and mortality. From a health system perspective, there is a need to enhance oncology service capacity, improve referral pathways, and strengthen primary healthcare systems to support early diagnosis and continuity of care. Collectively, these measures are essential to address the rising cancer burden and improve equitable access to comprehensive oncology care in the region.

## Figures and Tables

**Table 1:** Integrated Descriptive and Trend Analysis of Cancer Types (April 2023–February 2025).

Cancer Type	Total Cases	Mean/Month	Min	Max	Pearson R	R^2^	B Coefficient	*p*-Value	95% CI for B	Trend Interpretation
Breast cancer	5763	250.6	120	793	0.301	0.091	0.028	0.163	[−0.012, 0.069]	Positive but not statistically significant trend
Cervical cancer	3616	157.2	21	268	0.619	0.383	0.053	0.002	[0.022, 0.083]	Strong and statistically significant increasing trend
Prostate cancer	1099	47.8	11	109	0.164	0.027	0.055	0.455	[−0.096, 0.207]	No statistically significant trend
Lung cancer	483	21.0	6	79	0.549	0.302	0.224	0.007	[0.069, 0.379]	Significant increasing trend
Oesophageal cancer	831	36.1	8	193	0.085	0.007	0.016	0.701	[−0.068, 0.099]	No statistically significant trend
Other cancers	4316	187.7	76	295	0.490	0.240	0.054			

**Table 2:** Descriptive statistics of cancer patients and oncology service utilization at NMAH (April 2023–February 2025).

Variable	Total (n)	Mean per Month	Minimum	Maximum
Total cancer cases	16,368	706.4	355	1039
Male patients	4552	196.2	74	336
Female patients	11,816	510.3	227	793
New cancer cases	2137	92.9	31	168
Follow-up visits	14,761	642.6	277	985
Chemotherapy sessions	3864	168.0	70	300
Hormone therapy sessions	893	38.8	22	91
Palliative care encounters	16,911	735.3	355	1111
Inpatient admissions	11,019	479.1	7	845
Referrals	2763	120.1	31	845
Deaths	62	2.7	0	7

## Data Availability

Available from the corresponding author on reasonable request.

## Abbreviations

NMAH	Nelson Mandela Academic Hospital
ACS	American Cancer Society
ASCO	American Society of Clinical Oncology
HPV	Human Papillomavirus
LMICs	Low- and Middle-Income Countries
